# Increased primaquine total dose prevents *Plasmodium vivax* relapses in patients with impaired CYP2D6 activity: report of three cases

**DOI:** 10.1186/s12936-021-03869-x

**Published:** 2021-08-14

**Authors:** Anielle de Pina-Costa, Ana Carolina Rios Silvino, Edwiges Motta dos Santos, Renata Saraiva Pedro, José Moreira, Gabriela Liseth Umana, Ana Danielle Tavares da Silva, Otília Helena Lupi da Rosa Santos, Karina Medeiros de Deus Henriques, Cláudio Tadeu Daniel-Ribeiro, Patrícia Brasil, Tais Nobrega Sousa, André M. Siqueira

**Affiliations:** 1 Laboratório de Pesquisa Clínica em Doenças Febris Agudas–Instituto Nacional de Infectologia (INI) Evandro Chagas, Fundação Oswaldo Cruz (Fiocruz), Rio de Janeiro, RJ Brazil; 2 Centro de Pesquisa Diagnóstico e Treinamento em Malária–Fiocruz, Rio de Janeiro, Brazil; 3 Centro Universitário Serra Dos Órgãos (UNIFESO), Teresópolis, RJ Brazil; 4 Centro de Pesquisa Rene Rachou–Fiocruz, Belo Horizonte, Brazil; 5 Programa de Pós-Graduação Em Pesquisa Clínica, INI Evandro Chagas, Fiocruz, Rio de Janeiro, Brazil; 6 Assessoria Clínica–Instituto em Tecnologia em Imunobiológicos–Fiocruz, Rio de Janeiro, Brazil; 7 Laboratório de Pesquisa Em Malária, Instituto Oswaldo Cruz, Fiocruz, Rio de Janeiro, RJ Brazil

**Keywords:** *Plasmodium vivax*, Relapses, CYP2D6, Primaquine, Radical cure

## Abstract

**Background:**

The relapsing nature of *Plasmodium vivax* infection is a major barrier to its control and elimination. Factors such as adequate dosing, adherence, drug quality, and pharmacogenetics can impact the effectiveness of radical cure of *P. vivax* and need to be adequately evaluated. CYP2D6 pathway mediates the activation of primaquine (primaquine) into an active metabolite(s) in hepatocytes, and impaired activity has been linked to a higher risk of relapse.

**Cases presentation:**

Three patients diagnosed with *P. vivax* malaria presented repeated relapses after being initially treated with chloroquine (25 mg/kg) and primaquine (3.5 mg/kg in 14 days) at a non-endemic travel clinic. Recurring episodes were subsequently treated with a higher dose of primaquine (7 mg/kg in 14 days), which prevented further relapses in two patients. However, one patient still presented two episodes after a higher primaquine dose and was prescribed 300 mg of chloroquine weekly to prevent further episodes. Impaired CYP2D6 function was observed in all of them.

**Conclusion:**

Lack of response to primaquine was associated with impaired CYP2D6 activity in three patients presenting multiple relapses followed in a non-endemic setting. Higher primaquine dosage was safe and effectively prevented relapses in two patients and should be further investigated as an option in Latin America. It is crucial to investigate the factors associated with unsuccessful radical cures and alternative therapeutic options.

## Background

*Plasmodium vivax* is the most geographically widespread species causing human malaria, with approximately 40% of the world’s population at risk of infection [1–3]. There were nearly 157,000 new cases in Brazil in 2019, primarily due to *P. vivax* (89.1%), of which around 21% were classified as recurrences within 60 days [4]. Relapses accounted for around 33,000 episodes that year and earlier gametocyte production make this species particularly challenging for treatment and control, but the mechanisms leading to activating hypnozoites remain unknown [5].

The radical cure of vivax malaria requires anti-malarial drugs that target both blood and liver stages. Primaquine is the most available drug to eliminate hypnozoites [6, 7]. Primaquine’s clinical effectiveness is limited by the toxicity and potential haemolytic adverse events in patients with glucose-6 phosphate deficiency (G6PDd); that is why the drug is contraindicated during pregnancy and for infants less than six months.

There is no definitive method to differentiate recurrences of *P. vivax* as recrudescence (especially with increasing evidence of resistance to chloroquine [8]), relapses, and reinfection in areas with active transmission. The possibility of following patients in non-endemic areas provides an advantage where at least reinfection could be excluded. Recent studies in Brazil showed that recurrences rates range from 29.4 to 39.6% in Amazon and non-Amazon areas [9–11], despite primaquine’s routine prescription. It was recently described that the cytochrome P450 2D6 (CYP2D6) pathway mediates the activation of primaquine into active phenolic metabolite(s) in hepatocytes [12, 13] and some genetic polymorphisms implied in reduced primaquine metabolism have been associated with a higher risk of relapse [14–17]. Individuals with specific CYP2D6 polymorphic alleles fail to metabolize primaquine and may experience treatment failure, leading to false primaquine efficacy and tolerance assumptions.

CYP2D6 gene is highly polymorphic with over 150 alleles categorized in no, decreased, normal, and increased function alleles based on enzyme activity [18]. The CYP2D6 allele combinations give rise to different predicted metabolizer phenotypes: poor (gPM), intermediate (gIM), normal (gNM), and ultrarapid (gUM) metabolizers [18]. Therefore, vivax malaria patients with the defective CYP2D6 function would be at increased risk for therapeutic failure (relapses) regardless of proper treatment regimens with primaquine [14, 19, 20]. To identify patients at a higher risk for recurrences and their CYP2D6 metabolizer status, we describe three cases of multiple vivax malaria relapses in individuals with impaired CYP2D6 metabolic activity followed up at a non-endemic area in Brazil. The individual responses to different drug schemes varied related to CYP2D6 metabolic status and showed to be complex.

## Site and standard procedures

The Instituto Nacional de Infectologia Evandro Chagas (INI/Fiocruz) is a reference center for diagnosing and treating infectious diseases at Fundação Oswaldo Cruz, in Rio de Janeiro, Brazil. Patients with suspicion of malaria are evaluated by infectious disease physicians and follow the national malaria treatment guidelines. The guidelines state that vivax malaria should be treated with chloroquine (chloroquine) (25 mg/kg during three days) and primaquine (3.5 mg/kg during seven or 14 days). Blood slides were collected by experienced microscopists and malaria species confirmed by polymerase chain reaction (PCR) [21, 22]. Patients were followed until parasitological clearance and routinely at days 3, 7, 14, 21, 28, 40, and 60 post-treatment and at any time in case of recurring fever. All patients were tested for G6PDd. primaquine was adjusted for body weight (bw) when necessary. None of the patients returned to the endemic area.

### *CYP2D6* genotyping

Genotyping of one tri-nucleotide deletion (2615-2617delAAG [rs5030656]), eight single-nucleotide polymorphisms (SNPs) (− 1584C > G [rs1080985], 100C > T [rs1065852], 1023C > T [rs28371706], 1846G > A [rs3892097], 2850C > T [rs16947], 2988G > A [rs28371725], 3183G > A [rs59421388], 4180G > C [rs1135840]) and *CYP2D6* copy number analysis were performed by real-time PCR, according to protocols previously described [19, 23]. CYP2D6 haplotypes were inferred from genotypes using the software PHASE v.2.1 [24, 25] and phenotypes were predicted based on activity score (AS) model [18]. Patients were categorized into five predicted phenotype classes: poor metabolizer (gPM; AS score = 0), intermediate metabolizer (gIM; AS score = 0.5), normal-slow metabolizer (gNM-S; AS score = 1), normal-fast metabolizer (gNM-F; AS score 1.5–2.0), and ultrarapid metabolizer (gUM; AS score > 2).

Ethical approval was obtained from the INI/Fiocruz ethical review board (number 0020.0.009.000–07), and all participants provided informed written consent.

### Cases description

Herein, three cases of multiple *P*. *vivax* malaria recurrences are reported. All patients remained in the non-transmission area throughout the follow-up. Due to the lack of tools for differentiating relapses from recrudescence, criteria of classifying recurrences as either recrudescence or relapses were applied as following: recrudescence if happening less than 28 days post-treatment and relapses if occurring after this period. There was no risk of reinfection in the cases.

#### Case 1

**Fig. 1:**
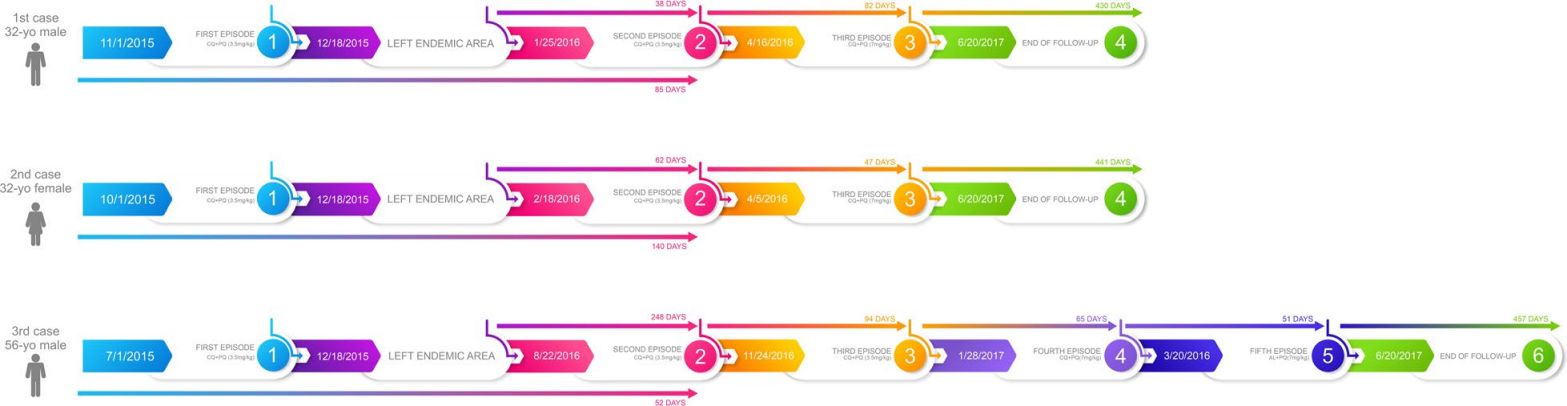
Dates and treatment regimens prescribed for the three cases

Male, 32 years old (yo), 78,5 kg of bw, resided in São Gabriel da Cachoeira (Amazon state) for two years (until 18th Dec 2015), where he had a diagnosis of vivax malaria on 1st Nov 2015 being treated with chloroquine and primaquine for seven days (Fig. 1). On 25th Jan 2016, 38 days after moving to Rio de Janeiro city (and 85 days after the initial diagnosis), he sought care at INI/Fiocruz and was diagnosed with *P. vivax* infection (18,320 parasites/mm^3^). G6PD activity was tested normal. He was treated with chloroquine and primaquine (total dose of primaquine: 3.44 mg base/kg given during nine days). On 16th Apr 2016 (81-days interval), he presented another malaria episode diagnosed as *P. vivax* (6,000 parasites/mm^3^). He was then treated with chloroquine and higher-dose primaquine (total dose of primaquine: 7.03 mg base/kg bw given in 22 days). CYP2D6 genotype was performed and classified as intermediate metabolizer (Table 1). He was followed up for more than one year and has not presented new episodes.

**Table 1:** Summary of CYP2D6 phenotype and enzymatic activity (activity score)

Patient	Allele 1	Allele 2	Activity score	CYP2D6 phenotype
Case 1	*9	*4	0.5	Intermediate metabolizer (gIM)
Case 2	*4	*4	0	Poor metabolizer (gPM)
Case 3	*41	*41	1.0	Normal-slow (gNM-S)

*CYP2D6 allele nomenclature according to PharmVar (https://www.pharmvar.org/gene/cyp2d6)

#### Case 2

Female, 32 yo, 62.3 kg of bw, resided in São Gabriel da Cachoeira (Amazon state) until 14th Nov 2015, where she was diagnosed and treated for vivax malaria on 1st Oct 2015 (Fig. 1). In Rio de Janeiro, she was diagnosed with *P. vivax* on 18th Feb 2016 (12,480 parasites/mm^3^) and was treated with chloroquine and primaquine (total dose of primaquine: 3.37 mg base/kg bw given in seven days). G6PD activity was tested normal. On 5th Apr 2016, she presented to INI/Fiocruz with a vivax relapse (46-days interval). She received chloroquine and primaquine (total dose of primaquine: 7.02 mg base/ kg bw given in seven days), and she remained without further episodes (updated 20th Jun 2017). CYP2D6 phenotype was classified as a poor metabolizer (Table 1).

#### Case 3

Male, 56 yo, 82 kg of bw, resided in Machadinho do Oeste (Rondônia state) for two months until 4th Aug 2016, where he received treatment for vivax malaria on 1st Jul 2016 (Fig. 1). After returning to Rio de Janeiro, he presented four episodes of malaria, with roughly similar intervals. For these episodes, the respective treatments were administered: chloroquine + primaquine (total dose: 3.21 mg/kg); chloroquine + primaquine (total dose: 3.21 mg/kg); chloroquine + primaquine (total dose: 7 mg/kg); Artemether-Lumefantrine (AL) + primaquine (total dose: 7 mg/kg)—AL was administered due to chloroquine-induced pruritus. CYP2D6 genotype was classified as a normal-slow metabolizer (Table 1). On 8th May 2017, after discussion with the patient, a decision to perform chloroquine prophylaxis (300 mg per week for eight weeks) was taken. After treatment with AL + primaquine and chloroquine prophylaxis, the patient was followed for more than one year and did not presented malaria recurrence.

## Discussion and conclusions

This study reports three individuals who presented a varied number of *P. vivax* relapses for which an impaired CYP2D6 activity was observed, suggesting that those abnormalities are implicated in the risk of *P. vivax* malaria recurrence after treatment with chloroquine/primaquine. The data reported here corroborate with a growing body of evidence that supports host genetics as an etiology of *P. vivax* relapse in individuals with primaquine failure. Whether routine screening of CYP2D6 alleles in patients who experience vivax malaria relapse in endemic settings is feasible and cost-effective is a matter that should be investigated. Next, more robust evidence is needed to identify the alternative treatment regimens in CYP2D6 impaired patients.

*Plasmodium vivax* is the most geographically widespread species causing human malaria, representing a challenge for control and elimination efforts mainly due to its complex biology [26]. The origin of a recurring parasitaemia following a primary infection by *P. vivax* can be a result of (i) recrudescence due to resistance to the blood schizonticidal drug – usually chloroquine; (ii) relapse from activated hypnozoites – which is a particularity of *P. vivax* and *Plasmodium ovale* amongst human malaria; or (iii) reinfection in areas where active transmission exist [7]. Relapses can be responsible for up to 80% of the malaria burden in given settings [27], suggesting that its relative contribution increases in declining transmission intensity [9]. The factors that trigger the hypnozoite activation are not completely understood and, strain-specific patterns, environmental factors, and host characteristics have been implicated as potential contributors. For the last six decades, primaquine, an 8-aminoquinoline derivative, has been the only drug with anti-relapse activity available. Its use was restricted due to the hemolytic potential in individuals with G6PDd [28]. Although the exact mechanism through which primaquine exerts its anti-relapse activity is unknown, recent findings that impaired CYP2D6 activity is associated with a higher risk of relapses point out the role of active metabolites produced by CYP2D6 against the parasite [14, 19].

There are no molecular methods to reliably distinguish amongst the causes of recurrence for *P. vivax* as there are for *P. falciparum.* Standardized molecular methods allow differentiation between recrudescence and reinfection [29]. All the relapses described in the three individuals occurred in a non-endemic area, and all subjects did not travel to any *P. vivax* endemic region after the initial episodes, thereby reducing the possibility that confounding variables were responsible for the observed relapse infection. Recrudescence due to erythrocytic parasites was not probable since the parasitaemia decreased in the blood, and therapeutic failure in the presence of the drug was not reported. The minimum interval between episodes was 52 days (median = 91, maximum = 136), which supports the classification of these recurrences as relapses since recrudescence due to erythrocytic stage parasites usually occur within 28 days after treatment with chloroquine [28].

Patients were oriented about the importance of treatment and reporting adverse events during the follow-up period, and returning to the clinic in case of symptoms. None of them returned to an endemic area and presented new symptoms after the therapeutic malaria period. The three subjects were tested negative for G6PDd, and the woman was not pregnant or breastfeeding. The results reported here corroborate partially with the study of Fernando et al. [30], indicating the use of higher total doses of primaquine to prevent relapses. Drug weight-adjusted during the treatment is essential, and all patients were treated with a high dose of primaquine. Two of them did not present relapses anymore. However, for one of the cases (case #3), a decision to institute weekly chloroquine prophylaxis was taken because relapses occurred even with primaquine in high doses. Of importance, none of the subjects had comorbidities or were using any non-antimalarial medication. Therefore, it is unlikely that host factors such as drug-drug and drug-CYP2D6 interactions influenced the pharmacokinetics and metabolism of primaquine by CYP2D6.

This study has some limitations. First, individual CYP2D6 phenotype was inferred from genotyping data, according to activity scores of CYP2D6 diplotypes, and there is evidence for a considerable range of variation in CYP2D6 function within genotype-inferred phenotype categories [31]. Second, although actual primaquine resistance could not be rule out, considering the early parasitological cure observed after the combined chloroquine/primaquine treatment, the most likely explanation is that the cause of the successive recurrences was due to primaquine failure and not primaquine resistance. Third, primaquine administration was not supervised, and the possibility of non-adherence may not be excluded. Nevertheless, all the patients reinforced that the total dosage of primaquine has been completed in all episodes, and all attended the follow-up appointments.

This case series, along with previous studies, points out that CYP2D6 is a possible important determinant of the efficacy of primaquine against relapse. A relevant issue for clinical management and, consequently, control and elimination is achieving a better radical cure and classifying and treating recurring episodes. Tafenoquine (TQ) has been licensed for malaria anti-relapse therapy and chemoprophylaxis by the U.S. Food and Drug Administration [32]. The significant advantage of TQ over primaquine is its long half-live of approximately 15 days allowing TQ to be administered in a single dose [33]. Although TQ is as haemolytic as primaquine [34], it is not clear how CYP2D6 polymorphisms impact drug efficacy for the radical cure of *P. vivax* malaria [35]. Evidence points to a more significant effect of CYP2D6 variants on primaquine metabolism; however, further investigations are required to determine the influence of poor metabolizers on TQ efficacy [36, 37]. Considering the burden of relapses and its public health implications for the elimination of vivax malaria in Latin America, a 1-year cohort, multicentre, therapeutic efficacy study of chloroquine and primaquine in distinct malaria transmission intensity locations in Brazil is being conducted to estimate the frequency, timing, and associated risk factors for the developing of recurrences (ABRACAMAL project, Gates’s foundation grant INV-003970). Thus, we aim to provide a comprehensive framework for an estimate the radical curative failure rate and thereby contribute to an improved understanding of the biology, epidemiology, and treatment of *P. vivax* malaria that may lead to more effective management policies.

## Data Availability

All the study data are available under request.

## Abbreviations

AL: Artemether-lumefantrine
AS: Activity score
bw: Body weight
chloroquine: Chloroquine
CYP2D6: Cytochrome P450 2D6
Fiocruz: Fundação Oswaldo Cruz
G6PDd: Glucose-6 phosphate deficiency
gIM: Intermediate metabolizer
gNM: Normal metabolizer
gPM: Poor metabolizer
gUM: Ultrarapid metabolizer
INI: Instituto Nacional de Infectologia Evandro Chagas
PCR: Polymerase chain reaction
primaquine: Primaquine
SNPs: Single-nucleotide polymorphisms
yo: Years-old
